# Effects of agriculture production systems on nitrate and nitrite accumulation on baby-leaf salads

**DOI:** 10.1002/fsn3.1

**Published:** 2013-01-08

**Authors:** Alfredo Aires, Rosa Carvalho, Eduardo A S Rosa, Maria J Saavedra

**Affiliations:** 1 CITAB-Centre for the Research and Technology of Agro-Environmental and Biological Sciences, University of Trás-os-Montes and Alto DouroP.O. Box 1013, 5001-801 Vila Real, Portugal; 2 Agronomy Department, University of Trás-os-Montes and Alto DouroP.O. Box 1013, 5001-801 Vila Real, Portugal; 3 CECAV-Veterinary and Animal Science Research Center, University of Trás-os-Montes e Alto DouroP.O. Box 1013, 5001-801 Vila Real, Portugal; 4 Veterinary Science Department, University of Trás-os-Montes e Alto DouroP.O. Box 1013, 5001-801 Vila Real, Portugal

**Keywords:** Baby-leaf salads, foodborne toxic incidence, nitrates, nitrites, production system

## Abstract

Nitrate and nitrite are widespread contaminants of vegetables, fruits, and waters. The levels of these compounds are increased as a result of using organic wastes from chemical industries, domestic wastes, effluents, nitrogenous fertilizers, and herbicides in agriculture. Therefore, determining the nitrate and nitrite levels in biological, food, and environmental samples is important to protect human health and the environment. In this context, we set this study, in which we report the effect of production system (conventional and organic) on the accumulation of nitrates and nitrites in fresh baby-leaf samples. The average levels of the nitrate (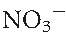) and nitrite (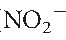) contents in six different baby-leaf salads of a single species (green lettuce, red lettuce, watercress, rucola, chard, and corn salad) produced in organic and conventional agriculture system were evaluated. Spectrophotometric analytical method recently published was validated and used. Nitrates and nitrites were detected in all samples. The nitrates levels from organic production varied between 1.45 and 6.40 mg/kg fresh weight (FW), whereas those from conventional production ranged from 10.5 to 45.19 mg/kg FW. The nitrites content was lower than nitrates and ranged from 0.32 to 1.89 mg/kg FW in organic production system and between 0.14 and 1.41 mg/kg FW in conventional production system. Our results showed that the nitrate content was dependent on the agricultural production system, while for nitrites, this dependency was less pronounced.

## Introduction

Western countries are consuming “organically” grown foods with increasing quantity and frequency (Jensen et al. 2011). The term organic production is applied to unprocessed agricultural crop products or minimally processed foods, which have not received excessive amounts of chemicals such as synthetic pesticides and fertilizers, without genetically modified organisms (GMOs) (EU 1995; Luttikholt 2007). Organic food is a small but increasing part of the food industry in the European Union. In Portugal, a recent study from Agriculture Ministry (GPP 2011) reported an increase in organic production. The total area dedicated to organic production was 210,981 ha in 2010 against 120,729 ha in 1994; in relation to vegetable sector, in 2010, the area occupied by organic mode was 737 ha against no more than 163 ha in 1994 (GPP 2011).

Organic production, as an alternative to conventional agricultural, relies on the incorporation of organic material in soil, normally by the use of animal manure as fertilizer (EU 1995). Animal manure is a good source of macronutrients and micronutrients, particularly nitrogen. The presence of nitrates is one of the consequences of the mechanism in which plants absorb the nitrogen element, in the form of 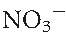, from fertilizers or organic materials (Gangolli et al. 1994), which are essential for the process of protein synthesis. Nitrates and nitrites are natural constituents of plant material, and they are normally present in high levels, particularly in green vegetables (Correia et al. 2010). Also, despite nitrate being an important component of plant material, it has the potential to accumulate in tissues, and thus, nitrate from fertilizers could accumulate in vegetables in large scale. Therefore, keeping nitrate concentrations below legal limits is a constant struggle for producers and farmers (Katan 2009). Nitrate is nontoxic below maximum residue levels (MRLs), but if it reaches above this level, it could be dangerous due to its reduction in nitrites, which can react with amines and amides to produce “N-nitroso” compounds responsible for gastric cancer (Santamaria 2006; Savino et al. 2006). High levels of nitrates in children stomach are responsible for methemoglobinemia (blue baby syndrome) (Greer and Shannon 2005; Chan 2011). Several factors influence the accumulation of nitrates in plants, including lack of sunlight or water, variety, maturity, high levels of fertilizers, nitrate levels in the soil, and quality of irrigation water. Excessive use of nitrogen fertilizers should be avoided so as to reduce nitrate buildup in soil and vegetables (Santamaria 2006). In order to maximize the health benefits from eating vegetables, measures should be taken to reduce the nitrate and nitrite exposures (Correia et al. 2010). Vegetables must be stored and processed properly to prevent bacterial contamination and hence reduction of nitrate to nitrite (Leszczyńska et al. 2009).

The preference for organic products is increasing all over Europe, due to the absence of chemical contaminants within this mode of production (EC 1991; EU 1995). In principle, organic products should contain fewer nitrates than their counterparts from conventional methods (Woese et al. 1997; González et al. 2010); however, some authors (De Martin and Restani 2003; Guadagnin et al. 2005) showed that content of nitrate in vegetables could be independent from the agricultural production system, and often organic vegetables could present very high nitrate average levels. Also many studies have demonstrated that organically grown crops have similar levels of nitrates and nitrites to their conventionally counterparts, and therefore doubts still persist. Pussemier et al. (2006) and Rembialkowska (2007) have published studies in which these contrasts are discussed. Therefore, despite of this growing interest in organic production, there is insufficient information to state categorically that the risk of nitrate or nitrite accumulation in organic production does not differ significantly from the risk associated with conventional practices. The results from different studies are inconsistent and doubts still persist. Thus, studies that can evaluate the growing conditions on the levels of nutrients and toxicants are urgently required. Therefore, the main objectives of this study were to assess the information on the nitrate and nitrite average levels on six different fresh baby-leaf salads produced and largely marketed in Northern Portugal, and to assess and determine the effect of production system (conventional and organic) on their accumulation in nitrates and nitrites and to estimate the toxicological risk associated with the consumption of baby-leaf salads containing nitrate and nitrite. The main aim of this study is to verify whether there is significant difference in nitrate or nitrite loads between organically and conventionally cultivated salads and whether such influence may increase the risk of disease occurrence for consumers.

## Material and Methods

### Sample collection

Samples of fresh baby-leaf salads were randomly acquired from supermarket (conventional production) and directly from local farmers (organic production), in Northern Region of Portugal, and included green leaf lettuce (*Lactuca sativa* var. *romana*), red leaf lettuce (*Lactuca sativa* var. *romana*), and watercress (*Nasturtium officinale* R. Br., *Brassicaceae*). Additionally, we extended this work on rucola (Rocket) (*Eruca sativa*; syn. *E. vesicaria* subsp. *sativa* (Miller) Thell., *Brassica eruca* L.), chard (*Beta vulgaris* var. cicla L.), and corn salad (*Valerianella locusta* L.), but only on samples from a conventional production system, due to the lack of this kind of vegetables in local organic farmers. The vegetables selected for this study are some of the most commonly consumed vegetables all over the year. All the commercial samples were obtained in original package, within the shelf life of up to 8 days, as declared on labels. After purchase, the samples were transported to the laboratory of phytochemicals at University of Trás-os-Montes e Alto Douro (UTAD) and 150 g were taken and freeze-dried (Dura-Dry™, μP-FTS Systems, NY), grounded in a blender (model BL41, Waring Commercial, Torrington, CT), and weighed. The dry matter was determined.

### Nitrate and nitrite analysis

The nitrate and nitrite contents in the vegetables were determined by a spectrophotometric method on foodstuff and water after zinc reduction and Griess reaction (Merino 2009). This method is based on the principle in which nitrate (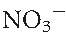) is reduced quantitatively to nitrite (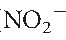) in the presence of zinc powder (Zn). The nitrite (that originally present plus reduced nitrate) is determined by diazotizing with sulfanilamide and coupling with *N*-(1-naphthyl)-ethylenediamine dihydrochloride to form a highly colored azo dye that is measured at 540 nm. The nitrite present in the sample is determined by analyzing without the reduction step. The nitrate is calculated as the difference between the total nitrite content after reduction and the initial nitrite concentration (Merino 2009). Three replicates were analyzed. The nitrate and nitrite levels were expressed as mg/kg fresh weight (FW).

### Statistical analysis

SPPS for windows version 17.0 (SPSS Inc., Chicago, IL) was used for statistical analysis. All experiments were performed in triplicate, and the results were presented as the mean ± SEM (standard error of the mean). The data were analyzed using one-way analysis of variance (ANOVA). The differences between the mean values were separated using Duncan's test at a significant level of *P* < 0.05.

## Results and Discussion

### Method validation and analytical quality assurance

A single-laboratory validation was carried out following the standard protocol adopted in the laboratory. The following parameters were evaluated: linearity, working range, detection and quantification limits, precision, recovery, and accuracy. Calibration curve was plotted, and linearity was evaluated by the values of determination and variation coefficients of the method after the application of several statistical tests. The calibration curves were obtained using a series of nitrate and nitrite standard solutions. All calibration curves were linear with correlation coefficients from the linear regression ranging from 0.992 to 0.999. Method performance data for nitrate and nitrite determination in baby-leaf salads are shown in Table 1. SPSS for windows version 17.0 and JMP windows version 7.01 (JMP SAS Institute, Cary, NC) were used to calculate all statistical parameters (means, standard deviations, coefficient of variation, minimum and maximum, correlation coefficient), and a *t*-test was used for determination of significant differences between the mean values.

**Table 1:** Method performance data

Analyte	Matrix	LOD (mg/kg)	LOQ (mg/kg)	Recovery range (%)	RSD_r_ (*n* = 6) (%)	Measurement uncertainty (%)	Accreditation (Yes/No)
NO_3_	Baby-leaf salad (lettuce)	1.2	1.4	73–105	3.30	7.7	No*
NO_2_	Baby-leaf salad (lettuce)	0.1	0.1	70–110	14.2	22	No*

* The method used for nitrate and nitrite determination is published in *Food Analytical Methods Journal* by Merino (2009).

### Nitrate contents of baby-leaf salads

To our knowledge, this is the first report that compares the nitrates/nitrites levels of fresh and baby-leaf salads in two types of agriculture. Individual results obtained for nitrate and nitrite levels are shown in Tables 2 and 3.

**Table 2:** Nitrate and nitrite concentrations in baby-leaf salads from organic and conventional farming in Northern Portugal.^1^,^2^

Plant material	Agriculture production system	[ ] Nitrites (mg/kg FW)	[ ] Nitrates (mg/kg FW)
Green lettuce	Conventional	0.25 ± 0.05a	26.05 ± 2.09b
	Organic	0.32 ± 0.13a	6.40 ± 1.48a
Red lettuce	Conventional	1.41 ± 0.11a	45.19 ± 4.54b
	Organic	1.89 ± 0.02b	5.16 ± 2.26a
Watercress	Conventional	0.81 ± 0.19a	42.76 ± 7.19b
	Organic	0.93 ± 0.01a	1.45 ± 0.30a

1 Values are expressed as mean ± SEM (standard error of the mean) of three replications.

2 Numbers with different letters in the same column, within same type of sample, are significantly different (*P* < 0.05), according to Duncan's test.

**Table 3:** Nitrate and nitrite concentrations in baby-leaf salads from conventional farming in Northern Portugal.^1^,^2^

Plant material	[ ] Nitrites (mg/kg FW)	[ ] Nitrates (mg/kg FW)
Rucola (Rocket)	0.18 ± 0.02a	17.82 ± 3.84b
Chard	3.48 ± 0.30b	23.13 ± 6.77b
Corn salad	0.14 ± 0.02a	10.57 ± 1.189a

1 Values are expressed as mean ± SEM (standard error of the mean) of three replications.

2 Numbers with different letters in the same column are significantly different (*P* < 0.05), accordingly to Duncan's test.

The results showed a considerable significant variation in the average levels of nitrate contents between the two production systems. The average levels of nitrates were higher (*P* < 0.05) in conventional produce, and as expected, the averages levels of nitrites were lower when compared with nitrates. The average levels of nitrites, except red lettuce, were very similar (*P* > 0.05) in both organic and conventional agriculture system (Tables 2 and 3). It seems that only the nitrate levels were significantly affected by the type of production. Similar tendency was found by Pussemier et al. (2006), who reported significant differences in the average levels of nitrate contents from organic and conventional produce. They reported lower levels of nitrates in organic (1703 mg/kg) and higher in conventional (2637 mg/kg) produces. Also, our findings showed a nitrate variation with plant family being the *Asteraceae* (lettuce) and *Brassicaceae* (watercress) – the families with the highest average levels. This result is in agreement with Santamaria (2006), who stated that families like *Brassicaceae* (rocket, radish, mustard and cress), *Chenopodiaceae* (beetroot, Swiss card, spinach), *Asteraceae* (lettuce), and *Apiaceae* (celery, parsley) are usually, among the vegetables, the plant families with highest nitrate contents. This tendency was confirmed in the present study.

The limits detected for nitrate in our samples are within the legal limits (<3000 mg/kg FW for lettuce and similar samples) recommended by European Union regulations (Regulation [EC] No. 1258/2011); thus, from the point of view of nitrates, this type of vegetables are safe. Moreover, the average content of nitrates is very far from those presented by Mor et al. (2010), which makes them very interesting from nutritional perspective. These plant materials can be used safely in adults, but also in infant meals, which oblige very low levels of nitrates and nitrites (Greer and Shannon 2005; Chan 2011).

One important aspect particularly to human health is related to contamination with nitrites. It is well accepted that when nitrates is reduced to nitrites, nitrite may react with amines or amides to form carcinogenic compounds (Savino et al. 2006). With regard to nitrite content of baby-leaf salads studied, our results showed a variation between 0.14 mg/kg FW for corn salad and 1.89 mg/kg FW for red lettuce. Moreover, only in the red lettuce, significant differences in nitrite contamination between organic and conventional produce (*P* < 0.05) were observed. For green lettuce and watercress, there were no significant differences. Nevertheless, the average nitrite content was higher in organic produce. Compared with literature, our average values are very similar to those reported in fresh vegetables (González et al. 2010; Mor et al. 2010). It is commonly assumed that the nitrite levels in fresh leafy vegetables are usually less than 2 mg/kg FW (Santamaria 2006). In this study, nitrite levels, except in chard, were lower than 2 mg/kg FW and very lower than the limits considered toxic (EU 1995).

Chung et al. (2004) and Prasad and Chetty (2008) have demonstrated that well-storage conditions are necessary to maintain the nitrites in low concentrations, due to minor activity of the enzyme reductase, responsible for reduction of nitrates in nitrites, and/or microbiological reduction of nitrates into nitrites. Under refrigerated storage, the nitrite accumulation tends to be reduced or even totally inhibited (Prasad and Chetty 2008). It seems to be the case of the samples studied in the current study. In fact, a poor storage must be avoided; otherwise, it results in bacterial growth, which can contribute to the increasing accumulation of high nitrite levels. Nitrate or even nitrite accumulation is dependent not only of agriculture system and respective practices but also of soil properties, fertilizer usage, cultivation, weather conditions, harvesting time, and size of vegetables and storage conditions (Tamme et al. 2006), which are unknown and whose effects are impossible to account in this study.

To conclude, based on our results, it seems that baby-leaf salads produced in organic and conventional systems in Northern Portugal have low levels of nitrates and nitrites. Therefore, they are toxicologically safe, and their consumption might be incremented without risk for health. The differences between organically and conventionally cultivated plants are less but exist. Nevertheless, it does not represent any type of risk for human health. These results are important not only for adults but particularly for children, in which the toxicological aspects related with nitrates and nitrites accumulation assume more importance.

## References

[b1] Regulation (EC) No. 1258/2011 (2011).

[b2] Chan TYK (2011). Vegetable-borne nitrate and nitrite and the risk of methaemoglobinaemia. Toxicol. Lett..

[b3] Chung JC, Chou SS, Hwang DF (2004). Changes in nitrate and nitrite content of four vegetables during storage at refrigerated and ambient temperatures. Food Addit. Contam..

[b4] Correia M, Barroso A, Barroso MF, Soares D, Oliveira MBPP, Delerue-Matos C (2010). Contribution of different vegetable types to exogenous nitrate and nitrite exposure. Food Chem..

[b5] EC (1991).

[b6] EU Scientific Committee for Food (1995).

[b7] Gabinete do Planeamento e Politicas (2011). http://wwwgpppt/Biologica/.

[b8] Gangolli SD, Van den Brandt PA, Feron VJ, Janzowsky C, Koeman JH, Speijers GJA (1994). Assessment of nitrate, nitrite and N-nitroso compounds. Eur. J. Pharmacol. Environ. Toxicol. Pharm..

[b9] González MCM, Martínez-Tomé MJ, Isasa MET (2010). Nitrate and nitrite content in organically cultivated vegetables. Food Addit. Contam..

[b10] Greer FR, Shannon M (2005). Infant methemoglobinemia: the role of dietary nitrate in food and water. American Academy of Pediatrics Committee on Nutrition, American Academy of Pediatrics Committee on Environmental Health, 2005. Pediatrics.

[b11] Guadagnin SG, Rath S, Reyes FGR (2005). Evaluation of the nitrate content in leaf vegetables produced through different agricultural systems. Food Addit. Contam..

[b12] Jensen KD, Denver S, Zanoli R (2011). Actual and potential development of consumer demand on the organic food market in Europe. NJAS – Wageningen J. Life Sci..

[b13] Katan MB (2009). Nitrate in foods: harmful or healthy?. Am. J. Clin. Nutr..

[b14] Leszczyńska T, Filipiak-Florkiewicz A, Cieślik E, Sikora E, Pisulewski PM (2009). Effects of some processing methods on nitrate and nitrite changes in cruciferous vegetables. J. Food Compos. Anal..

[b15] Luttikholt LWM (2007). Principles of organic agriculture as formulated by the International Federation of Organic Agriculture Movements. NJAS – Wageningen J. Life Sci..

[b16] De Martin S, Restani P (2003). Determination of nitrates by a novel ion chromatographic method: occurrence in leafy vegetables (organic and conventional) and exposure assessment for Italian consumers. Food Addit. Contam..

[b17] Merino L (2009). Development and validation of a method for determination of residual nitrite and nitrate in foodstuffs and water after zinc reduction. Food Anal. Methods.

[b18] Mor F, Sahindkuyucu F, Erdogan N (2010). Nitrate and nitrite content of some vegetables consumed in South province of Turkey. J. Anim. Vet. Adv..

[b19] Prasad S, Chetty AA (2008). Nitrate-N determination in leafy vegetables: study of the effects of cooking and freezing. Food Chem..

[b20] Pussemier L, Larondelle Y, VanPeteghem C, Huyghebaert A (2006). Chemical safety of conventionally and organically produced foodstuffs: a tentative comparison under Belgian conditions. Food Control.

[b21] Rembialkowska E (2007). Quality of plant products from organic agriculture. J. Sci. Food Agric..

[b22] Santamaria P (2006). Nitrate in vegetables: toxicity, content, intake and EC regulation (review). J. Sci. Food Agric..

[b23] Savino F, Maccario S, Guidi C, Castagno E, Farinasso D, Cresi F (2006). Methemoglobinemia caused by the ingestion of courgette soup given in order to resolve constipation in two formula-fed infants. Ann. Nutr. Metab..

[b24] Tamme T, Reinik M, Roasto M, Juhkam K, Tenno T, Kiis A (2006). Nitrates and nitrites in vegetables and vegetable-based products and their intakes by the Estonian population. Food Addit. Contam..

[b25] Woese K, Lange D, Boess C, Bögl KW (1997). A comparison of organically and conventionally grown foods – results of a review of the relevant literature. J. Sci. Food Agric..

